# Meibomian Gland Morphology Changes After Cataract Surgery: A Contra-Lateral Eye Study

**DOI:** 10.3389/fmed.2021.766393

**Published:** 2021-11-29

**Authors:** Pingjun Chang, Shuyi Qian, Zhizi Xu, Feng Huang, Yinying Zhao, Zhangliang Li, Yun-e Zhao

**Affiliations:** ^1^Eye Hospital and School of Ophthalmology and Optometry, Wenzhou Medical University, Wenzhou, China; ^2^National Clinical Research Center for Ocular Diseases, Wenzhou, China; ^3^Eye Hospital of Wenzhou Medical University, Hangzhou Branch, Hangzhou, China; ^4^Hangzhou Xiaoshan Liuliqiao Hospital, Hangzhou, China

**Keywords:** meibomian gland morphology, meibomian gland dysfunction, dry eye disease, meibomian gland atrophy, cataract surgery, self-contrast study, automated algorithm

## Abstract

**Purpose:** To evaluate the morphology changes of meibomian glands (MGs) after cataract surgery.

**Setting:** Hangzhou Branch of the Eye Hospital of Wenzhou Medical University, Zhejiang, China.

**Methods:** In this contra-lateral eye study, 40 patients received unilateral cataract surgery for age-related cataract. All the patients underwent the evaluation of non-invasive break-up time (NIBUT) and lower tear meniscus height (TMH) before the surgery and 6 months post-operatively. The MGs were evaluated via ImageJ and Meibomian Gland Bio-image Analyzer. MG dropout, length, width, area, gland diameter deformation index (DI), and gland signal index (SI) were recorded.

**Results:** MG length, width, area, DI, and SI were significantly decreased after cataract surgery in the study group (operated eyes, *P* < 0.001, *P* = 0.003, *P* < 0.001, *P* = 0.001, and *P* < 0.001, respectively) and showed no significant changes in the control group (non-operated eyes) (all *P* > 0.05). MG loss increased more in the study group (*P* = 0.030), and the changes in TMH and NIBUT were not significantly different between the two eyes (both *P* > 0.05).

**Conclusion:** Cataract surgery aggravated meibomian gland morphology, such as MG loss, MG length, width, area, and SI, and produced no change in NIBUT and TMH at 6 months post-operatively.

## Introduction

Phacoemulsification with intraocular lens implantation is a standard procedure for the treatment of cataract. However, the post-operative visual acuities were improved, but a number of patients often complained about dry eye symptoms. Meibomian gland dysfunction (MGD) is the main cause of evaporative dry-eye disease, which leads to tear lipid deficiency and tear film instability (1, 2). The previous studies reported that cataract surgery is associated with high incidence of MGD (3, 4).

Several studies have investigated MGD after cataract surgery; however, few have evaluated specific morphological changes (4–7). Han et al. (4) reported that cataract surgery aggravated MG function but had no effect on the MG structure. MG morphology was evaluated by the grade of MG loss, and no quantitative parameters for each gland were assessed in their study. Nevertheless, a number of studies have demonstrated the relationship between MG morphology changes and MGD (5, 8). By analyzing the morphological changes using optical coherence tomography meibography (OCT-M), Liang et al. (8) revealed that MG length and width were decreased in the patients with MGD. The morphological changes of the meibomian gland (MG), such as shortening, dilation, distortion, and atrophy are sensitive and early indicators of MGD (9, 10).

In recent years, infrared non-contact meibography, confocal microscopy, and optical coherence tomography meibography are introduced to precisely visualize the MG structure. Keratograph 5M (K5M, Oculus, Wetzlar, Germany) is the most widely used infrared non-contact meibography for clinical MG morphology image acquisition (11).

Other factors, such as aging, sex, environmental factors, poor sleep quality, and sleeping position are associated with MGD (12–15), thus it is necessary to include the unoperated contralateral eye as the control when assessing the influence of cataract surgery. The previous studies did not involve the contralateral eyes as a control group to exclude confounding factors.

To our knowledge, this is the first contra-lateral study to evaluate the MGD and morphological changes in the patients who underwent cataract surgery. We analyzed multiple parameters offered by a new automated algorithm (16) to estimate the function and morphology of the MGs. Additionally, we explored the association between the MG structure and clinical tests in these patients.

## Patients and Methods

### Patients

This study enrolled patients who underwent unilateral cataract phacoemulsification with intraocular lens implantation at the Hangzhou Branch of the Eye Hospital of Wenzhou Medical University from May 2017 to October 2019. The operated eyes were enrolled as the study group and the contra-lateral eyes as the control group. All the procedures followed the tenets of the Declaration of Helsinki. The ethics approval was obtained from the Ethics Committee of the Eye Hospital, Wenzhou Medical University, Wenzhou, China (ID: 2020-058-K-52).

**Figure 1:**
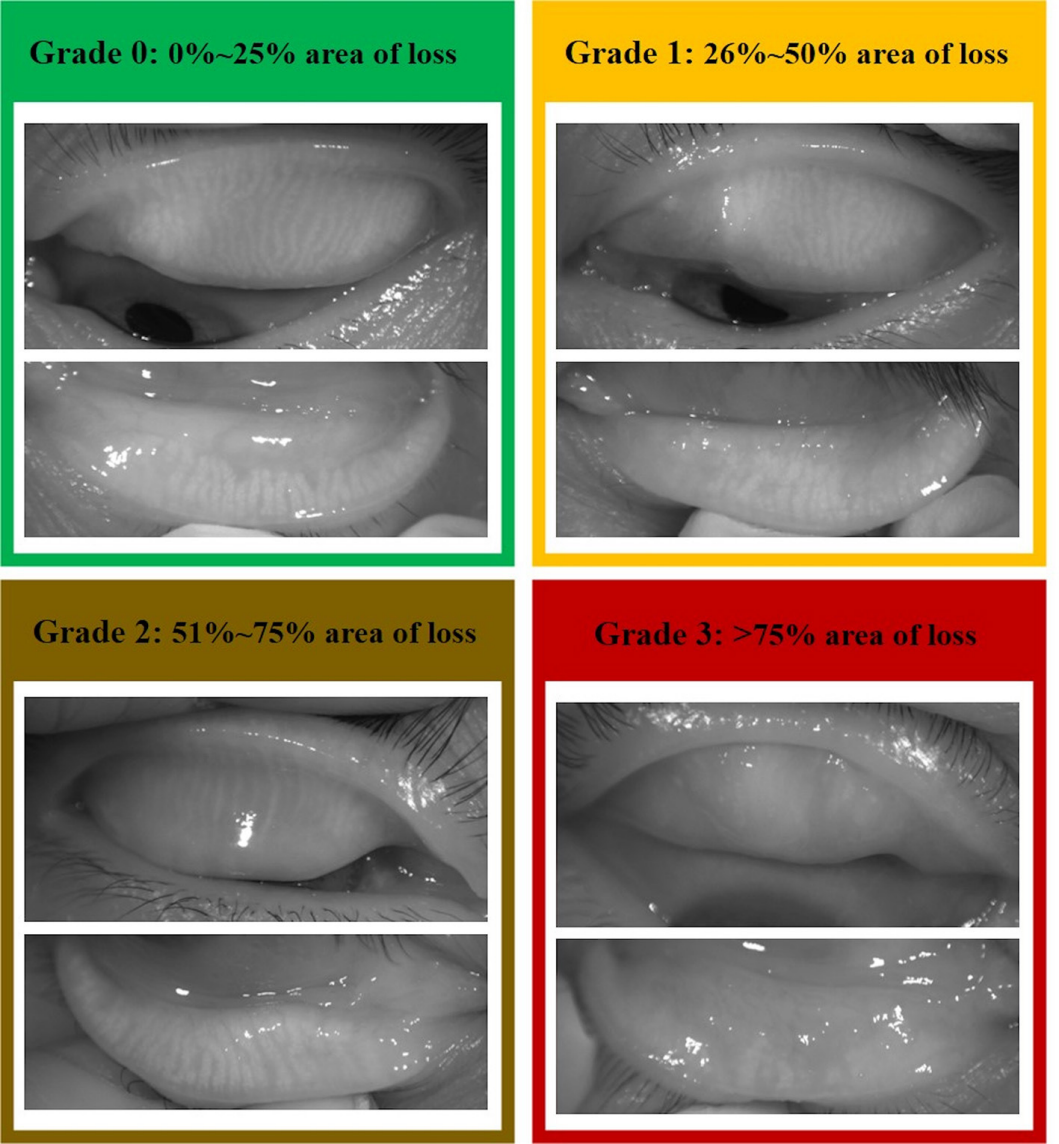
The meibograde grading system reported by Adil et al. was referred to do subjective grading of meibomian gland (MG) loss.

The patients who had a similar degree of MG atrophy in both the eyes with grade 1 or 2 MG loss (defined as the proportion of the area of meibomian gland in its relation to the total area of the tarsus) were included (Figure 1). The patients who had previous ocular diseases, history of ocular surgery or contact lens wear, and who used topical or systemic medications that may affect the ocular surface were excluded.

### Cataract Surgery

All surgeries were performed by a single surgeon (Zhao YE). The conjunctival sac was irrigated with 5% Povidone-iodine. Topical anesthesia was administered with 0.5% proparacaine hydrochloride eye drops. After capsulorhexis and phacoemulsification through a 2.2 mm incision, a 1-piece intraocular lens was placed in the capsular bag. At the end of surgery, the corneal incision was sealed with stromal hydration.

Post-operatively, all the patients were prescribed 0.5% topical levofloxacin (Cravit; Santen Pharmaceutical, Osaka, Japan) four times daily for 2 weeks and tobramycin-dexamethasone eye drops (Tobradex^®^, Alcon Couvreur NV, Puurs, Belgium) four times daily for the first week and tapered for the following 3 weeks.

### Parameter Collections

Meibography was performed using a Non-contact Meibography System (Kerotography 5M, Oculus, Wetzlar, Germany) within 5 days before the operation and at ~6 months after the operation.

**Figure 2:**
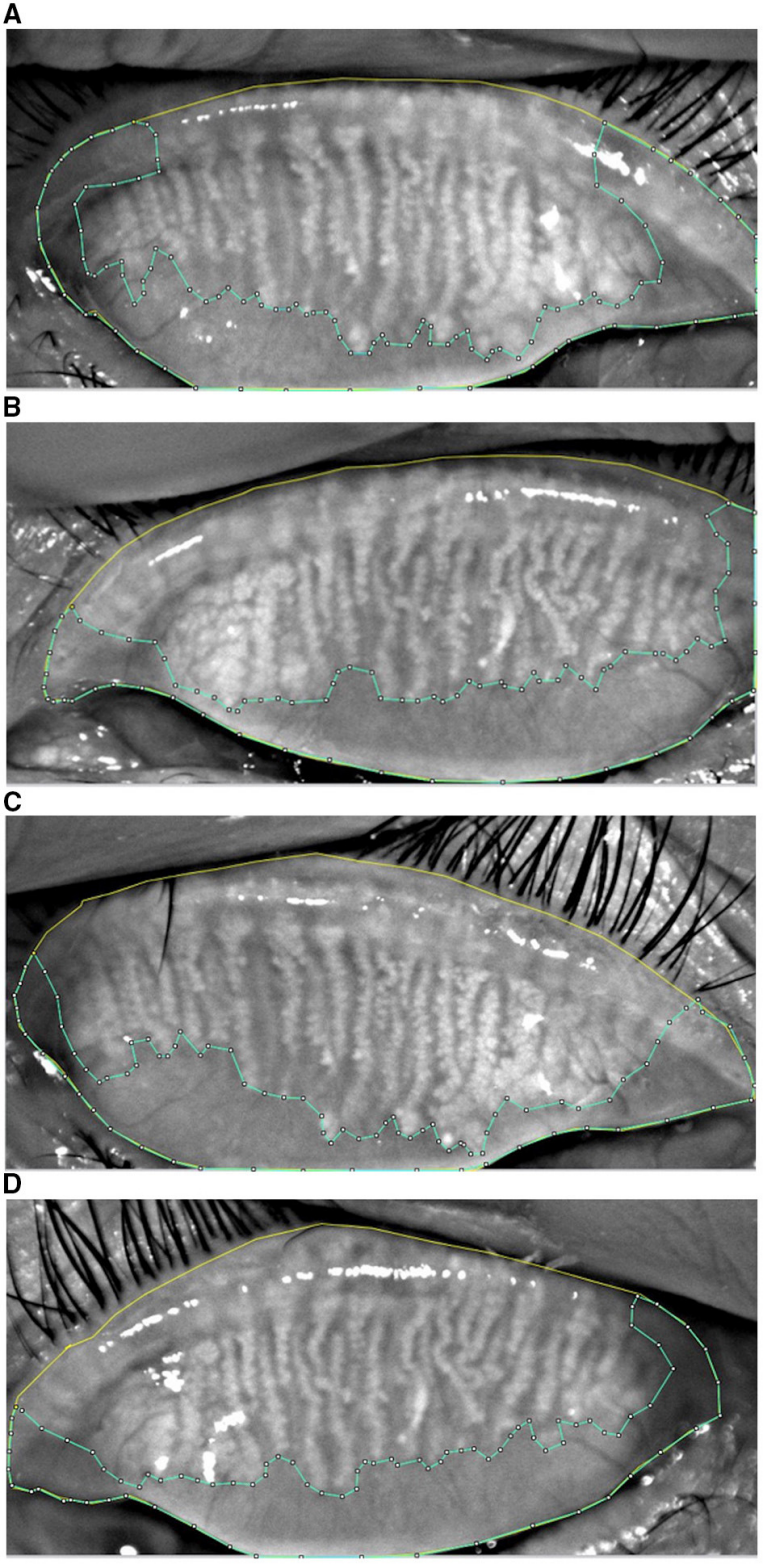
The upper eyelid (yellow) and the edge of MG loss (blue) was drawn in the MG morphology images acquired from K5M. MG loss was calculated by the ImageJ software in the study group at baseline **(A)**, the control group at baseline **(B)**, the post-operative study group **(C)**, and the post-operative control group **(D)**.

The MG loss area was calculated using ImageJ software (National Institute of Health; http://imagej.nih.gov/ij, Figure 2). Meibograde grading scheme reported by Adil et al. (9) was referred to evaluate the MG loss: grade 0, 0–25% area of loss; grade 1, 26–50% area of loss; grade 2, 51–75% area of loss; and grade 3, >75% area of loss (Figure 1).

**Figure 3:**
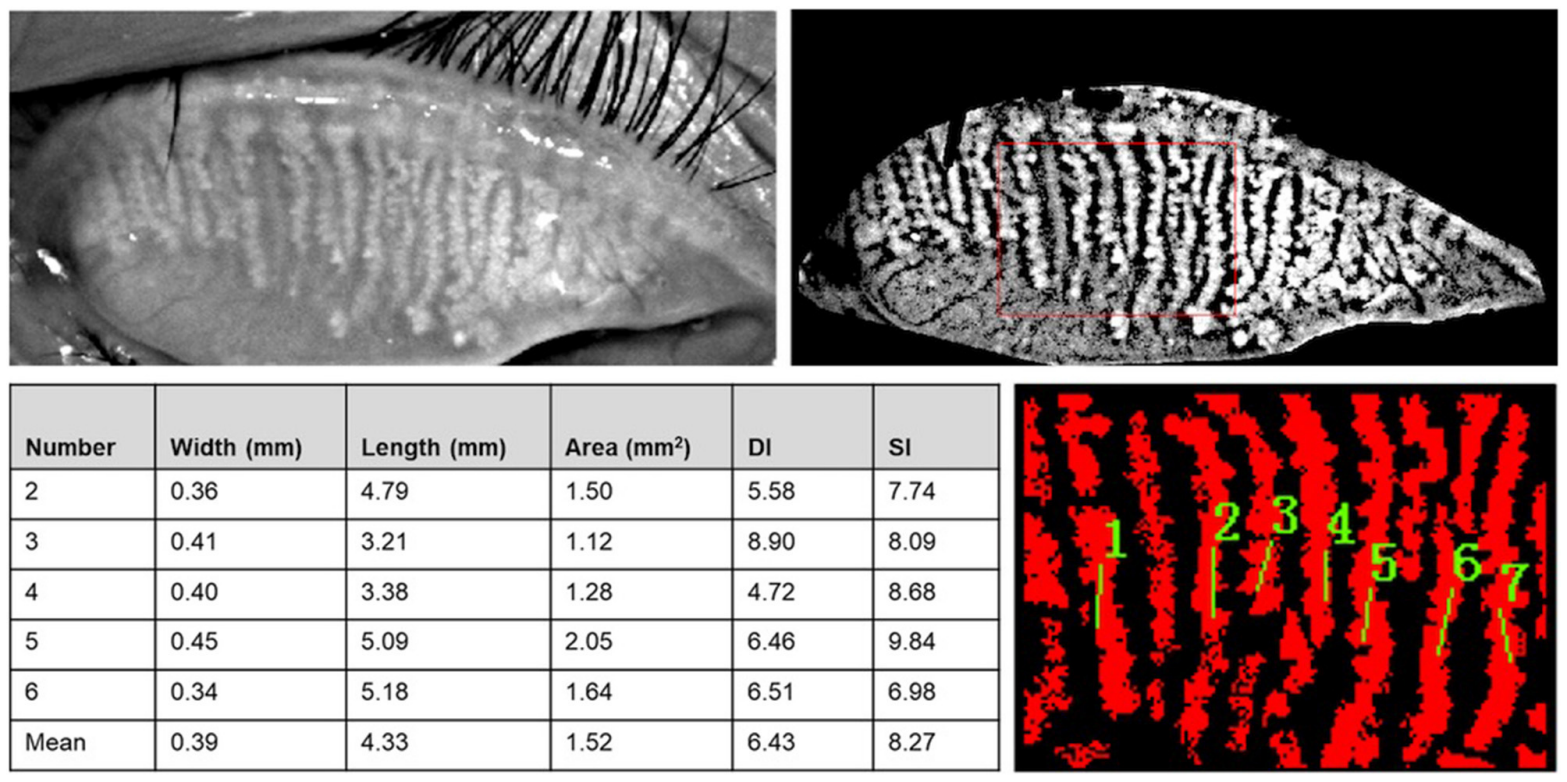
Meibomian Gland Bio-image Analyzer V3 software dealt with images from K5M and dampened the random noises and highlighted the visibility of glands. The width, length, area, DI, and SI of central five MGs were obtained after choosing the central part of the image and marked the MGs.

MG parameters, such as length, width, area, gland diameter deformation index (DI), and gland signal index (SI) were analyzed using Meibomian Gland Bio-image Analyzer V3 software (16) (Figure 3) on the basis of the meibography images acquired by Kerotography 5M (K5M). This new automated algorithm developed by Xiao et al. offered multiple parameters for objective evaluation of MG morphology. The algorithm consists of three main steps: (I) region of interest (ROI) acquisition; (II) segmentation and identification of MGs within the ROI; and (III) quantitative parameter analysis. The five central MGs of upper lid were adopted to measure the length, width, area, DI, and SI changes of the MGs.

Additionally, the tear meniscus height (TMH) and the average non-invasive break-up time (NIBUT) was measured by K5M. The measurements were performed three times, and the average of the three attempts was used for analyses.

### Statistical Analysis

The normal distribution of the data was verified using the Shapiro–Wilk test. The differences between the operated eye and contralateral eye, as well as the differences between the pre-operative and post-operative parameters, were assessed using the paired-samples *t*-test or Wilcoxon signed-rank test according to the data normality. Spearman's rank correlation coefficient was used to assess the correlations between the changes in TMH, NIBUT, and MG morphology in each group. The statistical analyses were performed using SPSS for Windows version 26.0 (IBM Corp., NY, USA). The criterion for statistical significance was *P* < 0.05.

## Results

### Demographics

A total of 80 eyes of 40 patients (25 men and 15 women) were included in this study. The mean age of the patients was 71.20 ± 9.73 years (range 38 to 87 years). The mean MG loss was 35.05 ± 12.56% in the study group (meibograde 1: 28 eyes; meibograde 2: 12 eyes) and 36.29 ± 12.78% in the control group (meibograde 1: 30 eyes; and meibograde 2: 10 eyes), respectively (*P* = 0.278, Table 1). At baseline, no significant differences were found between the two groups in TMH, NIBUT, and MG parameters except for MG length (the study group: 4.15 ± 1.09 versus the control group: 3.72 ± 0.89, *P* = 0.017, Table 1).

**Table 1:** The changes in parameters before and at 6 months after cataract surgery in both groups.

	**Baseline**			**6 months**			***P*** **_study_**	***P*** **_control_**	***P*** **_Δ_**
	**Study group**	**Control group**	***P*** **_base_**	**Study group**	**Control group**	***P*** **_6 mon_**			
MG loss (%)	35.05 ± 12.56	36.29 ± 12.78	0.278	37.84 ± 12.43	37.82 ± 12.56	0.984	0.004	0.015	0.030
MG length (mm)	4.15 ± 1.09	3.72 ± 0.89	0.017	3.64 ± 0.85	3.76 ± 0.92	0.466	<0.001	0.698	0.001
MG width (mm)	0.48 ± 0.14	0.49 ± 0.11	0.706	0.43 ± 0.09	0.50 ± 0.16	0.001	0.003	0.694	0.007
MG area (mm^2^)	1.67 ± 0.65	1.53 ± 0.47	0.204	1.36 ± 0.53	1.59 ± 0.64	0.035	<0.001	0.506	0.002
DI	8.60 ± 4.36	9.40 ± 3.54	0.242	6.89 ± 2.94	9.97 ± 5.32	<0.001	0.001	0.717	0.004
SI	5.36 ± 1.48	5.56 ± 1.44	0.351	4.60 ± 1.28	5.53 ± 1.14	<0.001	<0.001	0.846	<0.001
TMH (mm)	0.25 ± 0.13	0.23 ± 0.11	0.209	0.26 ± 0.14	0.26 ± 0.13	0.518	0.460	0.032	0.685
NIBUT(s)	6.76 ± 4.35	6.57 ± 3.64	0.658	6.51 ± 4.09	6.28 ± 4.10	0.360	0.555	0.492	0.736

*The paired-samples t-test was applied for normal distribution, and the Wilcoxon matched-pair signed rank test was applied for abnormal distribution. P_base_: The study group vs. the control group at baseline; P_6mon_: The study group vs. the control group at 6 months post-operatively; P_study_: The parameters at baseline vs. at 6 months post-operatively in the study group; P_control_: The parameters at baseline vs. at 6 months post-operatively in the control group; P_Δ_: Change of the parameters in the study group vs. that in the control group.*

*MG, Meibomian gland; DI, gland diameter deformation index; SI, gland signal index; TMH, tear meniscus height; NIBUT, non-invasive break-up time*.

### Changes in the Parameters After Cataract Surgery

As shown in Table 1, MG loss worsened in both the study group and the control group (*P* = 0.004 and *P* = 0.015, respectively). Despite similar MG loss were observed between baseline and post-operative in the two groups (both *P* > 0.05), we found that the change in MG loss was more severe in the study group (*P* = 0.03). Significant changes were observed after cataract surgery between the two groups in MG width, MG area, DI, and SI (*P* = 0.001, *P* = 0.035, *P* < 0.001, and *P* < 0.001, respectively). MG length, MG width, MG area, DI, and SI were significantly decreased after surgery in the study group (*P* < 0.001, *P* = 0.003, *P* < 0.001, *P* = 0.001, and *P* < 0.001, respectively) and showed no significant changes in the control group (all *P* > 0.05). The decrease in MG length, MG width, MG area, DI, and SI in the study group were significantly larger than those in the control group (*P* = 0.001, *P* = 0.007, *P* = 0.002, *P* = 0.004, and *P* < 0.001, respectively). However, no significant difference was found when comparing NIBUT, TMH, and the changes of these two parameters between the two groups at baseline and 6 months post-operatively. Moreover, NIBUT and TMH were not significantly changed in either group except that the control group displayed increased post-operative TMH values (*P* = 0.032)

**Figure 4:**
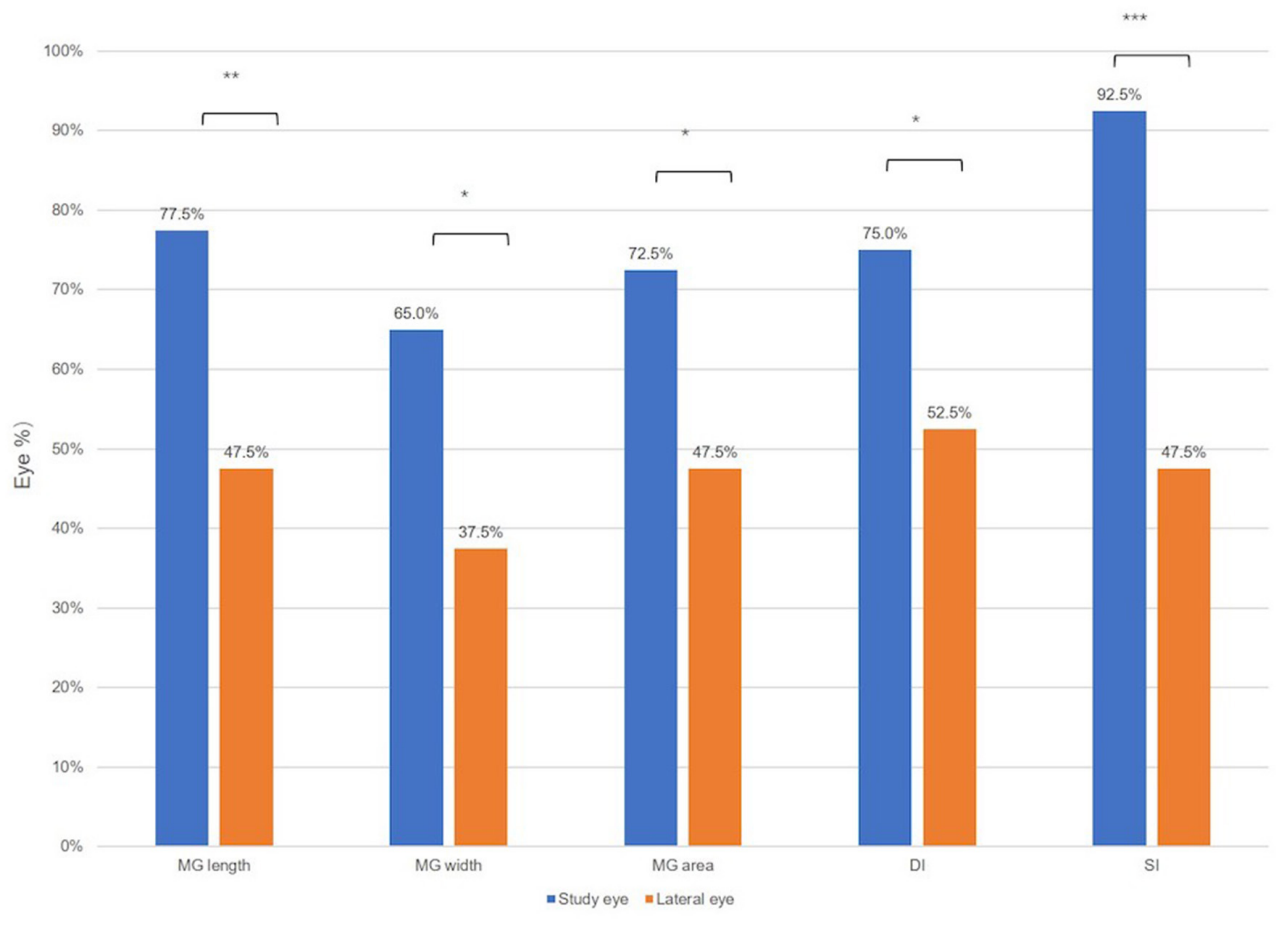
The proportion of eyes whose ocular surface parameters value were decreased after the cataract surgery. Wilcoxon signed-rank test: *P < 0.05, **P < 0.01, and ***P < 0.001.

As shown in Figure 4, the proportion of eyes whose parameter values decreased after cataract surgery. More eyes in the study group displayed decrease of MG width (65.0 vs. 37.5%, *P* = 0.012), MG length (77.5 vs. 47.5%, *P* = 0.003), MG area (72.5 vs. 47.5%, *P* = 0.033), DI (75.0 vs. 52.5%, *P* = 0.029), and SI (92.50 vs. 47.5%, *P* < 0.001) than those in the control group.

### Correlations Between NIBUT, TMH, and MG Morphology

**Figure 5:**
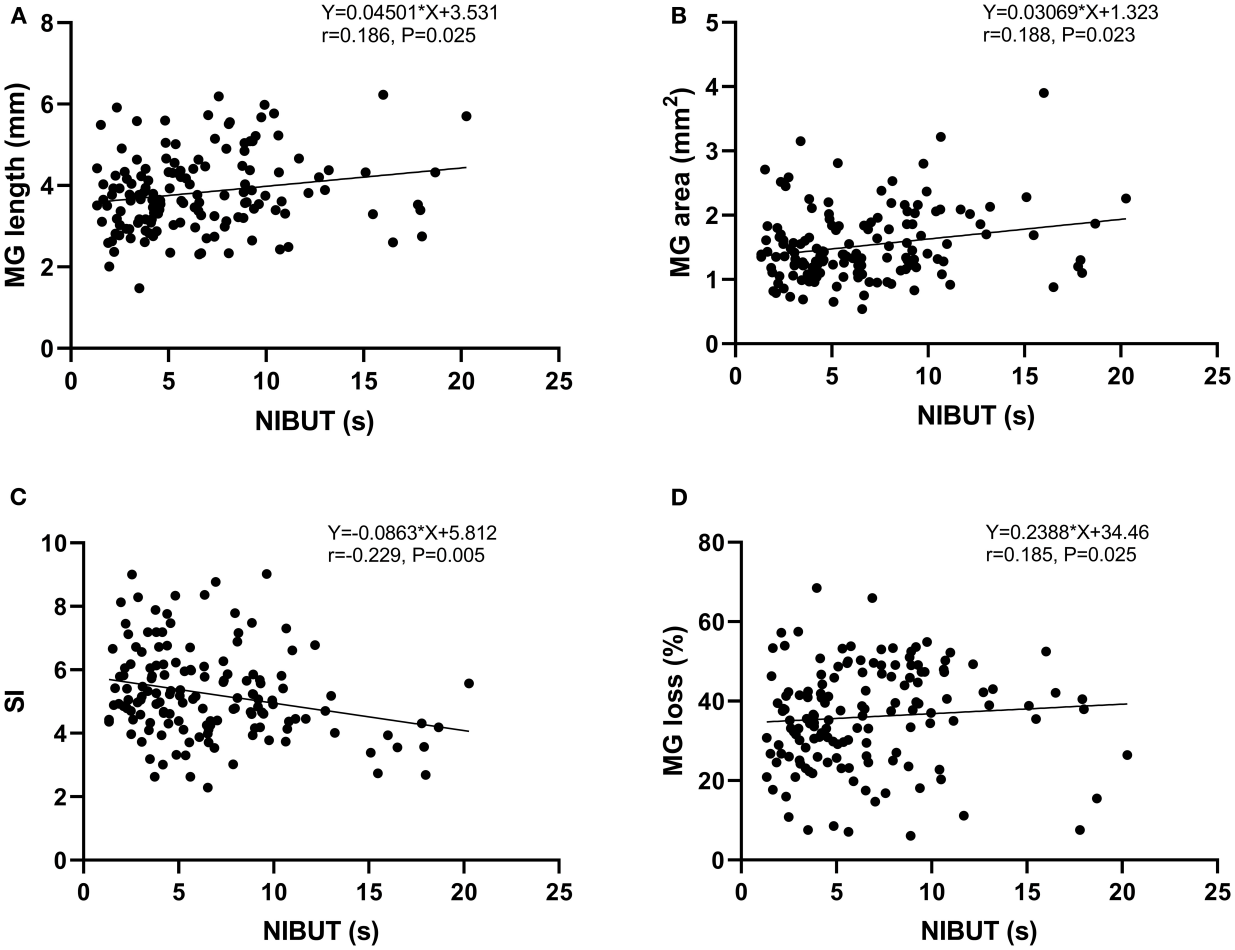
Analysis of the Spearman's rank correlation between the NIBUT and MG length **(A)**, MG area **(B)**, SI **(C)**, and MG loss **(D)**.

Spearman's rank correlation coefficient was used to assess the correlations. Table 2 showed there was a positive correlation between NIBUT and MG length (*r* = 0.186, *P* = 0.025), MG area (*r* = 0.188, *P* = 0.023), and MG loss (*r* = 0.185, *P* = 0.025), whereas a negative correlation between NIBUT and SI (*r* = −0.229, *P* = 0.005) (Figure 5). Additionally, a significant correlation was found between MG loss and TMH (*r* = 0.221, *P* = 0.005).

**Table 2:** Correlation coefficients of clinical tests and meibomian gland (MG) morphology.

	**TMH (mm)**		**NIBUT(s)**	
	***r***	***P***	***r***	***P***
MG length (mm)	−0.016	0.844	0.186	0.025
MG width (mm)	0.002	0.980	0.033	0.695
MG area (mm^2^)	−0.015	0.846	0.188	0.023
DI	−0.152	0.055	−0.016	0.849
SI	−0.097	0.221	−0.229	0.005
MG loss(%)	0.221	0.005	0.185	0.025

*TMH, tear meniscus height; NIBUT, non-invasive break-up time; MG, Meibomian gland; DI, gland diameter deformation index; SI, gland signal index*.

## Discussion

MGD is one of the most common ocular surface diseases, which aggravates dry eye symptoms and leads to dissatisfaction in people who undergo cataract surgery. We performed the clinical tests and meibography based on the K5M to observe the functional and morphological changes in the MG after cataract surgery. To obtain more reliable and repeatable results, a fully automated algorithm was used for the objective evaluation of meibography, and a contra-lateral comparison was designed to exclude other factors.

A previous prospective observational study reported that cataract surgery aggravated MG function without changing the structure (4). They analyzed the MG loss at 1 and 3 months after cataract surgery. However, we found MG loss aggravation at 6 months after cataract surgery. Different follow-up time, the sample size and analysis approach might be the probable cause of different outcomes. After discovering more MG loss post-operatively, we evaluated the details of MG structure for further analysis and observed meibomian glands deterioration 6 months post-operatively. Using the automated algorithm reported by Xiao P. et al. (16), it is possible to analyze and compare the details of MG structure, such as length, width, area, DI, and SI.

In the present study, after analyzing the central five glands in the upper lid, we observed significant decreases in MG length, width, area, DI, and SI in the study group. The parameters, such as length, width, and area described the general morphology of MG, the decrease of which further proved deterioration of MG loss assessed by ImageJ software. Gland irregularity was first defined as the differential area compared with a “standard gland” through an automated morphological method in a previous study (17). They verified that gland irregularity was a good parameter for predicting gland function. In this study, we used DI to quantify the uneven width of the MG and found decreased values in the operated eyes. The probable explanation was that the measuring range got smaller due to the decrease of MG area. SI quantified the gland signal level according to the secretion, consistency, and color of meibum inside meibomian glands, which can potentially be used to evaluate meibum secretion activity (16). The decrease in SI in the study group indicated less meibum secretion of MG after cataract surgery.

No significant difference was found between the two groups in MG loss at baseline or 6 months post-operatively. Interestingly, the contra-lateral group also showed MG loss aggravation 6 months post-operatively, slightly less than the study group (*P* = 0.015 vs. *P* = 0.004). The possible reason is that lack of eyelid cleaning in both eyes in the early post-operative stage may result in eyelid margin deterioration, and then result in meibomian glands loss. When comparing MG morphology, there was no difference in MG width, area, DI, and SI values at baseline, but the post-operative values showed significantly lower in the study group. MG length showed no difference between the two groups post-operatively, it may be due to the higher pre-operative MG length value in the study group. Furthermore, all the morphological parameters changed more in the study group than in the control group.

As previously reported, MGD is associated with higher MG loss (9). We investigated TMH and NI-BUT as the functional parameters in the present study and revealed no significant changes in long-term post-operative NIBUT and TMH compared with the pre-operative levels or the contralateral eyes. We found no significant difference when comparing the NIBUT and TMH between the two groups at baseline and 6 months post-operatively. Although TMH increased in the control group post-operatively, TMH changes were not significantly different between the two groups. No significant NIBUT changes were found in either group after cataract surgery. Being consistent to our results, a study by Choi et al. (5) reported no significant difference at 1 and 3 months after surgery in TBUT when compared with the baseline data. A recent study by Lin et al. (18) showed that there was no difference in TMH between the patients with MGD and normal controls. However, some studies reported that TBUT and TMH decreased 3 months post-operatively (4, 7, 19). Based on the observation of 192 eyes of 96 patients, Cetinkaya et al. (20) noted that phacoemulsification surgery may aggravate dry eye test values in the patients with chronic dry eye in the short term (1st day, 1st week, and 1st month post-operatively), and these values will return to the pre-operative levels in the long term (3rd month, 6th month, 1st year, and 2nd year post-operatively). The possible reason of the controversial viewpoints was that most of the studies followed up in the early post-operative periods of cataract surgery, for example, 1 or 3 months post-operatively, while we included the 6-month data after the surgery. We speculate that the NIBUT value gradually compensated and returned to the baseline level, thus at 6 months post-operatively, it displayed slightly shorter than that at baseline without statistically significance.

The MG morphology parameters showed some weak correlations with the clinical tests. There was a positive correlation between TMH and MG loss (*r* = 0.221, *p* = 0.005). Furthermore, there was a positive correlation between NIBUT and MG length (*r* = 0.186, *p* = 0.025), MG area (*r* = 0.188, *p* = 0.023) and MG loss (*r* = 0.185, *p* = 0.025), whereas a negative correlation between NIBUT and SI (*r* = −0.229, *p* = 0.005). Several researchers reported significant strong correlation coefficients (21–23). Hence, TMH and NIBUT could partly represent the function of mebomian glands, especially NIBUT. Theoretically, MG loss indicating MGD that is negatively correlated with NIBUT. It is controversial with our results, although it was weakly correlated. The reason might be as follows: clinically, MG loss does not necessarily mean the MGD if there are no dry eye symptoms. Furthermore, NIBUT may gradually return to the baseline level in the long term after surgery while the changes of MG were irreversible.

The present study reported that MG morphology was aggravated even at 6 months post-operatively, and revealed the morphological changes more specifically, filling the lack of long-term investigation. However, this study has several limitations. First, this study only included 6-month post-operative examinations, so it was unable to determine the changing process after cataract surgery. Second, the lid margin, meibum expressibility were not investigated in this study. Third, we did not perform the Ocular Surface Disease Index (OSDI) Questionnaire, refraction or visual acuity tests in these patients, these parameters and their correlations with MG morphology aggravation need further study in the future. What's more, although we evaluated gland morphology in detail, this study only enrolled central five glands in the upper lid, unable to conclude the morphological changes in all glands. Hence, it is necessary to conduct a study that includes more parameters and glands in the lower lid at several time points.

In conclusion, our study revealed that cataract surgery was associated with MG loss aggravation, and MG morphology deterioration, such as a decrease in length, width, area, and gland signal index. Post-operative NIBUT and TMH did not significantly change in the long term. Therefore, it is important for the clinicians to monitor the MG morphological parameters before and after cataract surgery.

## Data Availability Statement

The raw data supporting the conclusions of this article will be made available by the authors, without undue reservation.

## Ethics Statement

The studies involving human participants were reviewed and approved by the Ethics Committee of the Eye Hospital, Wenzhou Medical University (ID: 2020-058-K-52). The patients/participants provided their written informed consent to participate in this study.

## Author Contributions

PC and Y-eZ: study concept and design. SQ, ZX, and FH: data collection. PC, SQ, and YZ: analysis and interpretation of data. SQ and ZX: writing the manuscript. PC: critical revision of the manuscript. ZL: statistical expertise. Y-eZ: administrative, technical, or material support and supervision. All authors contributed to the article and approved the submitted version.

## Funding

This study was supported by research grants from the Science and Technology Program of Wenzhou City (Y2020369); the Medical and Health Technology Program of Zhejiang Province (2018KY538); and the National Natural Science Foundation of China (Grant No. 81870680).

## Conflict of Interest

The authors declare that the research was conducted in the absence of any commercial or financial relationships that could be construed as a potential conflict of interest.

## Publisher's Note

All claims expressed in this article are solely those of the authors and do not necessarily represent those of their affiliated organizations, or those of the publisher, the editors and the reviewers. Any product that may be evaluated in this article, or claim that may be made by its manufacturer, is not guaranteed or endorsed by the publisher.
